# Supplementary material for: Acidosis Activates the Nrf2 Pathway in Renal Proximal Tubule-Derived Cells through a Crosstalk with Renal Fibroblasts
Source: Antioxidants (Basel). 2023 Feb 8;12(2):412. doi: 10.3390/antiox12020412 (PMC9952787; doi:10.3390/antiox12020412)
Supplement: Supplementary file 1 [file antioxidants-12-00412-s001.zip › antioxidants-2124897-supplementary.pdf]

Supplementary figures

Supplementary figure S1

https://software.broadinstitute.org/morpheus/:

Clustering:
Eucledian distance
Linkage method = average

Similarity matrix:
Pearson correlation

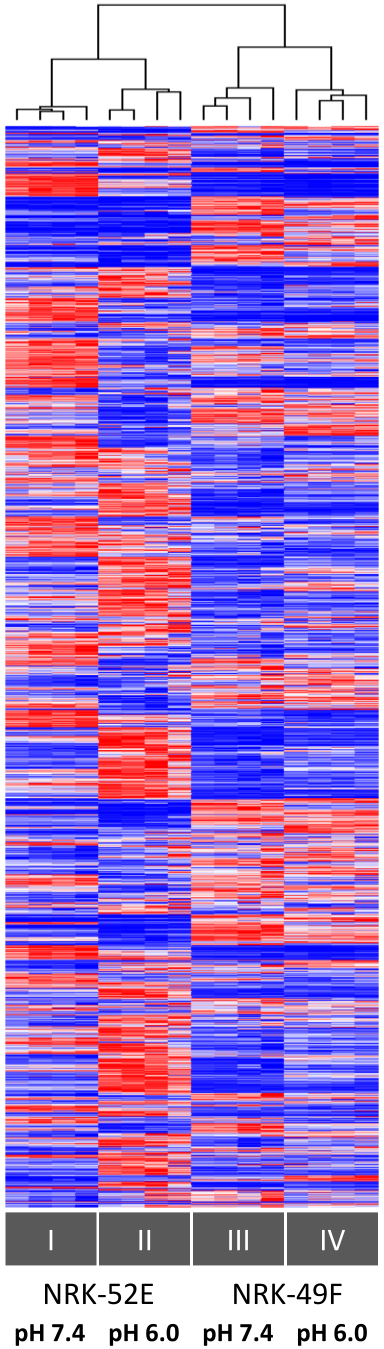

all, FPM>10, 11122 genes

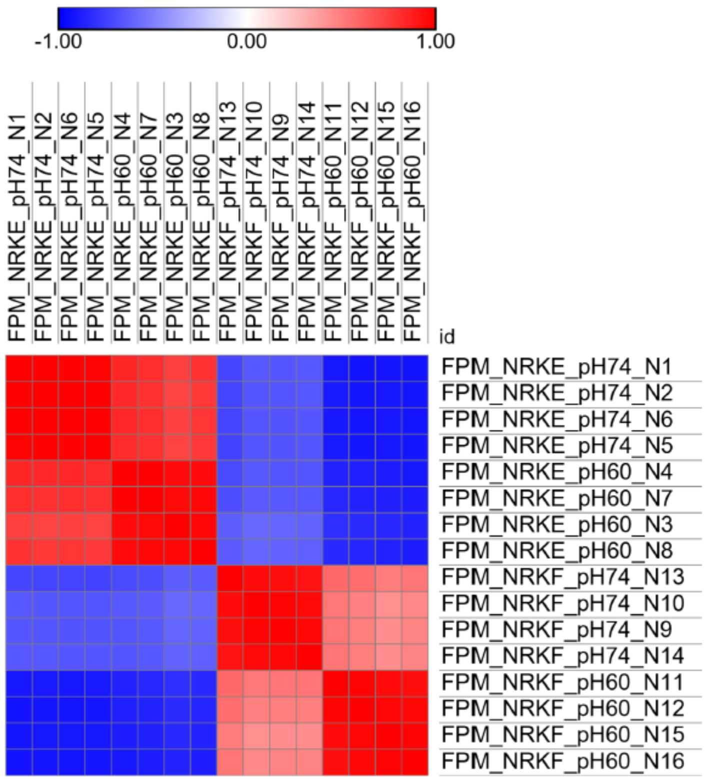

Supplementary Table S1. Buffer composition

EDTA buffer	• 136.8 mM NaCl<br>• 2.68 mM KCl<br>• 8.1 mM Na_2HPO_4 x 2 H_2O<br>• 2 mM KH_2PO_4<br>• 0,7 mM EDTA<br>• pH 7,2
MOPS-Triton buffer	• 10 mM TRIS base<br>• 20 mM MOPS<br>• 100 mM NaCl<br>• 1 mM EDTA<br>• 0.01 % Triton X-100<br>• pH adjusted to 7.5
6 x Redmix/Lämmli buffer	• 124.8 mM TRIS HCl (AppliChem GmbH, Darmstadt, GER)<br>• 6 % SDS (Carl Roth GmbH&CoKG, Karlsruhe, GER)<br>• 1,42 mM ß-Mercaptoethanol (Sigma-Aldrich Co, St. Louis, USA)<br>• 4,7 mM Glycerol (Sigma-Aldrich Co, St. Louis, USA<br>• 155.2 mM Bromphenol Blue (SERVA Electrophoresis GmbH, Heidelberg, GER)<br>• pH 6.8
running buffer	• 25 mM TRIS

	• 3,5 mM SDS<br>• 192 mM glycine
transfer buffer	• 20 % MeOH<br>• 25 mM Tris<br>• 192 mM glycine
trypsin solution	• 154 mM NaCl<br>• 2.7 mM KCl<br>• 8.2 mM Na_2HPO_4<br>• 1.5 mM KH_2PO_4<br>• 0,7 mM EDTA<br>• 0,2 mM streptomycin<br>• 0,18 mM penicillin<br>• 0.02 mM trypsin<br>• pH 7,1-7,3
1xTBS TWEEN	• 3 mM TRIS base<br>• 140 mM NaCl<br>• 0.17 mM TRIS-HCl<br>• 1 % TWEEN 20<br>• pH 7.4
equilibration buffer	• 10 mM TRIS (pH 7.3)

	• 10 mM KCl<br>• 1.5 mM MgCl<br>• 0.5 mM ß-Mercaptoethanol<br>• 1 : 100 protease inhibitor
lysis buffer	• equilibration buffer<br>• 0.4 % nonidet P40
extraction buffer	• 20 mM HEPES<br>• 0.4 M NaCl<br>• 1 mM EDTA<br>• 1 mM DTT<br>• 1 : 100 protease inhibtor

Supplementary Table S2. Primer sequences and annealing temperatures

Gene name	Accession number	Forward 5′-3′	Backward 5′-3′	Annealing °C
Hprt1	NM_012583.2	ACCAGTCAACGGGG-GACATA	TTGGGGCTG-TACTGCTTGAC	60 °C
Nrf2 (Nef2l2)	NM_031789.2	ACTACAGTCCCAG-CAGGACA	GTTTGG-GAATGTGGG-CAACC	60 °C
Tkt	NM_022592.2	TCCATACCATGCGC-TACAAG	ATAAGATGG-GAGCTGCATGG	60 °C

Abbreviations: *Hprt - Hypoxanthine Phosphoribosyltransferase 1, Nrf2 (Nef2l2) - Nuclear factor erythroid 2-related factor 2, Tkt - transketolase*
